# Annual Meeting of the Erie Co. Medical Society

**Published:** 1858-03

**Authors:** 


*Annual Meeting of the Medical Society of the County of Erie.*—The annual meeting of this society, was held in this city, on Tuesday, Jan. 12th, ult., at the Rooms of the Buffalo Medical Association, No. 7 South Division street. It was one of the largest, if not the largest, gathering of the members ever assembled. A goodly number of members were in from the country, and besides a number from neighboring counties were present by invitation.

The President, Prof. F. H. Hamilton, presided. The business of the session was commenced by the reading of the minutes of the last meeting by the secretary, Dr. James M. Newman.

The business of the day was limited entirely to the transactions of the ordinary business of the society: and no exciting questions were proposed, or discussed.

The usual annual report was received from the Librarian, Dr. Samo. An appropriation for the re-imbursement of the Library was recommended.

The Treasurer, Dr. Newman, reported

| | |
|---|---|
| The receipts for the year, . . . . . | $69 75 |
| Expenditures (including fifty-two dollars law expenses), . . . . . . . . | 84 00 |
| Expenditures over receipts, . | $14 25 |

The committee appointed at the special meeting, Nov. 22d, 1856, to employ counsel in behalf of the society to defend it in the suit brought by Dr. J. D. Hill,

*Reported*, That they had employed Messrs. Talcott & Thompson in the case, and that the cause had been argued before the Supreme Court, Judge Marvin, presiding; and that the society had been beaten, and a peremptory mandamus had been issued compelling the society to reïnstate Dr. Hill into all his privileges as a member of the society.

That the expenses attending the defense of the said suit had been fifty-two dollars.

A resolution was accordingly passed, restoring Dr. Hill to "to whatever and all the standing, right and privileges he had enjoyed in this society previous to the passage of the aforesaid resolution of expulsion."

The committee also reported, that an alternative mandamus had been sued out by Dr. E. P. Gray against the society, and as the points in the suit of Dr. Gray had been discussed and considered in the decision rendered in the case of Dr. Hill, and as the opinion of the court had been pronounced adversely to the society, they had not deemed it advisable, or of any avail to take steps to defend the society in the suit of Dr. Gray

A resolution of similar import to that of Dr. Hill, was then passed by the society in reference to Dr. Gray.

The annual Oration was then delivered by the secretary, Dr. James M. Newman: subject, "*Albuminuria, Independent of Organic Disease of the Kidneys.*" The paper will be found in another portion of this Journal.

Dr. J. S. Hawley, was appointed *Orator* for the semi-annual meeting, and Dr. B. H. Lemon, his substitute.

After the transaction of some business having reference merely to the duties and wants of the day, but of not sufficient public interest to report, the society proceeded to the annual election of officers, and made choice of the following gentlemen to fill the respective offices:

| | |
|---|---|
| Dr. Austin Flint, . . . . . | President. |
| " L. P. Dayton, . . . . . . | Vice-president. |
| " James M. Newman, . . . . | Secretaıy. |
| " John Root, . . . . . . | Treasurer. |
| " J. B. Samo, . . . . . | Librarian. |

*Primary Board.*—Dr. Sandford Eastman, J. S. Hawley, and J. Hauenstein.

| | | | |
|---|---|---|---|
| *Censors.*—Dr. B. H. Lemon, | Exam. | in Anat. | Physiology and Surgery. |
| " W. Gould, | " | " | Pract. Med. and Obstetrics. |
| " C. B. Hutchins, | " | " | Chemistry and Pharmacy. |
| " C. C. F. Gay, | " | " | Mat. Med. and Botany. |
| " L. J. Ham, | " | " | Med. Jurisp. and Pathology. |

*Delegates to the State Medical Society.*—Drs. C. H. Wilcox, John Boardman, P. H. Strong, and Wm. Van Pelt.

The President and Secretary were authorized to appoint Delegates to the American Medical Association.

The President, Prof. Hamilton, then delivered his *Valedictory Address.*

At its conclusion, a vote of thanks was tendered the speaker.

The business of the day being concluded, the society adjourned.

The members, however, shortly after convened again, at the American Hotel, where in one of the parlors, a session of some four hours was held in discussing the good things spread upon the table of the host, Mr. Hodges, and in the interchange of sentiments appropriate to the occasion. The season of convivial enjoyment furnished a fiitting close to the duties of the day; and were such occasions more frequent among the members of our profession, many of the asperities of feeling, and petty jealousies and heart burnings which too often mar the pleasures of professional intercourse would be wiped out and forgotten. N.

---

*The Secretory and Excito-secretory System.* By H. F. Campbell.

In our notice of the Transactions of the Americal Medical Association, we took pleasure in referring as fully as possible, to the excellent papers of Dr. Campbell. These have been collected by the author and elegantly bound in muslin, for a copy of which we tender him our thanks. F.

